# The inter-prefectural regional disparity of healthcare resources and representative surgical procedures in orthopaedics and general surgery: a nationwide study in Japan during 2015–2019

**DOI:** 10.1186/s12891-023-06820-0

**Published:** 2023-09-12

**Authors:** Masamitsu Kido, Kazuya Ikoma, Yumiko Kobayashi, Masahiro Maki, Suzuyo Ohashi, Katsutoshi Shoda, Daisuke Ichikawa, Ritei Uehara, Kenji Takahashi

**Affiliations:** 1 Department of Orthopaedics, Graduate School of Medical Science, Kyoto Prefectural University of Medicine, 465, Kajii-Cho, Kawaramachi-Hirokoji, Kamigyo-Ku, Kyoto, 602-8566 Japan; 2 Department of Rehabilitation Medicine, Graduate School of Medical Science, Kyoto Prefectural University of Medicine, Kyoto, Japan; 3 First Department of Surgery, Faculty of Medicine, University of Yamanashi, Yamanashi, Japan; 4 National Institute of Public Health, Saitama, Japan

**Keywords:** Epidemiology, Surgical procedure, Medical specialist, Medical facility, National database

## Abstract

**Background:**

Few reports have examined the localized regional disparity in representative surgical procedures in orthopaedics and general surgery globally. This study aimed to clarify the inter-prefectural regional disparity and relationships between healthcare resources and representative surgical procedures using a nationwide database in Japan.

**Methods:**

The number of medical specialists in orthopaedics, general surgery, and anaesthesiology, as well as the number of hospitals, and the incidence of representative surgical procedures in orthopaedics and general surgery were evaluated annually per 100,000 inhabitants/people by prefecture in Japan during 2015–2019. Medium-sized regional disparities were evaluated using the Gini coefficient. Correlation coefficients were calculated for the defined variables and ageing rate. We also compared the urban and rural regional disparities in all study variables.

**Results:**

The annual average number/incidence and Gini coefficients were 110.6 and 0.11 for femur fracture surgery, 106.3 and 0.09 for cholecystectomy, 14.2 and 0.11 for orthopaedic surgeon specialists, 17.6 and 0.09 for general surgeon specialists, 5.9 and 0.13 for anaesthesiology specialists, and 8.1 and 0.21 for hospitals, respectively. The correlation coefficients by the incidence of femur fracture surgery were 0.74 for orthopaedic surgeon specialists (*p* < 0.001), 0.63 for hospitals (*p* < 0.001), and 0.62 for the ageing rate (*p* < 0.001); those by the incidence of cholecystectomy were 0.60 for general surgeon specialists (*p* < 0.001) and 0.59 for hospitals (*p* < 0.001). The number/incidence of orthopaedic surgeon specialists, hospitals, femur fracture surgery, and cholecystectomy, as well as the ageing rate, were significantly higher in rural prefectures than in urban prefectures (*p* < 0.05).

**Conclusions:**

Inter-prefectural regional disparity was small, although certain items were unevenly distributed in the rural prefectures, which is contrary to our expectations. Higher prevalence was recognised in rural prefectures due to the higher ageing rate; however, supply and demand are balanced. This study provides basic data for healthcare policy development in a medium-sized community.

**Level of evidence:**

III.

**Supplementary Information:**

The online version contains supplementary material available at 10.1186/s12891-023-06820-0.

## Background

Japan is an island country in East Asia with a total area of approximately 380,000 km^2^ and a total population of approximately 125,000,000 (final estimates as of 1 February 2022 Statistics Bureau of Japan [1]), with the majority of individuals being Asian. According to World Bank data from 2021 (https://databank.worldbank.org/), Japan’s economy ranks third in the world by nominal gross domestic product (GDP) but 26th by nominal GDP per capita. The healthcare insurance system [2] is characterised by universal health coverage, allowing citizens to decide which medical facilities to visit, when and where needed, without financial hardship. The index of universal health coverage in Japan is one of the highest in the world [3]. Additionally, physicians can freely choose their departments and specialities. However, as in other countries [4, 5], the maldistribution of physicians to urban rather than rural areas has been an ongoing challenge [6–8]. In 2020, a limiting system (setting limits on the number of trainees in a prefecture for each department and allowing them to train in areas with a shortage of physicians) was launched to address the maldistribution.

The rapidly ageing population poses additional challenges to the healthcare system. In 2020, the ageing rate (the ratio of the elderly population aged ≥ 65 years to the total population) in Japan was the highest in the world at 28.7%. However, the ageing rate is expected to increase further by 2040, with a declining birth rate and a society with an ageing rate of 35.3% (https://www.stat.go.jp/data/topics/topi1261.html). As the working population declines, major changes in the quality and quantity of medical care are expected. Therefore, it is essential to understand the medical needs and demands of communities. In particular, surgical treatment is a common medical demand and a powerful tool for medical personnel to treat diseases. However, few reports have examined the localized regional disparity in representative surgical procedures globally.

Representative surgical procedures common within an ageing society in orthopaedics include osteosynthesis (fracture surgery) for acute trauma and arthroplasty (joint replacement) for chronic osteoarthritis. Recently, hip and knee arthroplasties have increased annually in Japan [9], while the occurrence of hip fractures is markedly increasing [10, 11]. Additionally, common general surgeries include cholecystectomy [12] and appendectomy [13]. Although an epidemiological survey of representative surgical procedures in general surgery [14], and some epidemiological studies on these representative surgical procedures have been reported in Japan, few reports have examined localized regional disparities in the world. Moreover, essential data related to the number of medical specialists and the incidence of surgical procedures are sparse worldwide [15] and the relationship between medical specialists, facilities, and representative surgical procedures have not been characterized using a large-scale database.

The National Database of Health Insurance Claims and Specific Health Checkups of Japan (NDB) contains most data (> 95%) regarding healthcare insurance claims for medical treatments, as monitored by the Ministry of Health, Labour, and Welfare [16, 17]. In fact, after complete anonymisation, > 1.7 billion records are annually registered in the NDB [17, 18]. Hence, the NDB is useful for evaluating nationwide surgical procedures.

We hypothesised that regional disparities in medical care are small across Japan, and healthcare resources and surgical procedures are correlated and concentrated in urban areas. Using the NDB, this study aimed to investigate the regional disparity and relationships among healthcare resources and representative surgical procedures in each medium-sized region (prefecture) in Japan.

## Methods

### Ethics statement

This study did not require institutional board approval or informed consent because of the use of legally anonymised public data.

### Study design and population

Using the NDB Open Data Japan [19], the annual average number/incidence of medical specialists, facilities, and surgical procedures were surveyed from 2015 to 2019 in 47 prefectures. The incidences of outpatient and inpatient surgeries were summed for each year, whereas the annual nationwide incident subjects of total surgical procedures were calculated over five years. The NDB guidelines stipulate that when the incidence is < 10, the data cannot be reported for anonymisation. Biennial data (2016, 2018) on the number of physicians by prefecture from the Ministry of Internal Affairs and Communications open data [20] were used to determine the number of medical specialists in orthopaedics, general surgery and anaesthesiology. The number/incidence per 100,000 inhabitants/people was calculated based on the demographics of the Ministry of Internal Affairs and Communications [20]. We also evaluated the number of hospitals per 100,000 inhabitants and beds per 1,000 inhabitants, using open data from 2017 [20]. A hospital was defined as a facility that can accommodate ≥ 20 patients. Correlation coefficients were calculated for surgical procedures, medical specialists, hospitals, and beds, by prefecture. From the top 10 highest-volume surgical procedures in orthopaedics in 2020 in Japan [21], we examined the following seven surgical procedures assigned K-codes as per the Japanese coding system for surgical procedures: five fracture surgeries (femur, forearm, lower leg, upper arm [codes K046 1, 2], and femoral head replacement [code K081 1]), and two arthroplasty surgeries (total hip arthroplasty [code K082 1], and total knee arthroplasty [code K082 1]). The remaining three spine surgeries were excluded as neurosurgeons and orthopaedic surgeons performed the surgeries in Japan. According to the Organization for Economic Co-operation and Development (OECD) criteria, hip replacement was calculated as the sum of total hip arthroplasty and femoral head replacement. In general surgery, major surgical procedures were examined as surveyed by the OECD: cholecystectomy (open [code K672]; laparoscopic [code K672-2]) and appendectomy (open [code K718]; laparoscopic [code K718-2]). The ageing rate in each prefecture was calculated from the demographics of the Ministry of Internal Affairs and Communications [20]. The Gini coefficient (0–1; 0: complete equality, 1: complete inequality) was also calculated according to previous reports [6, 22, 23] to examine regional disparity among prefectures. The Gini coefficient is an indicator of the degree of income inequality and other factors that has been widely used in the field of economics and to assess the distribution of physicians in a region. It is categorized as low (< 0.2), moderate (≥ 0.2, < 0.3), high (≥ 0.3, < 0.4), or extreme inequality (≥ 0.4).

### Prefectures and urban–rural definition

**Fig. 1:**
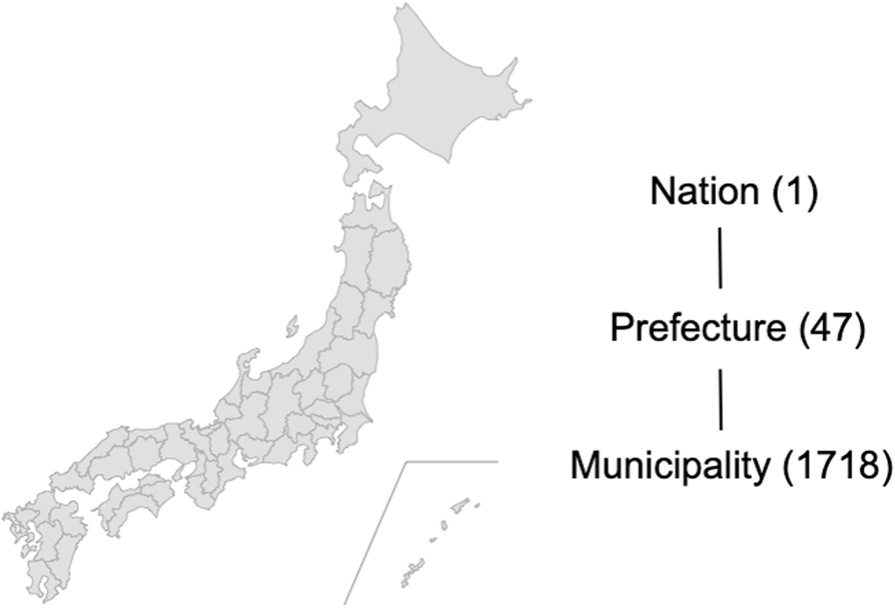
Map of Japan showing the 47 prefectures The Japanese government is regulated by a hierarchy of nations, prefectures, and municipalities. The country is divided into 47 prefectures and 1,718 municipalities

Japan has three administrative levels: the national government, prefectures, and municipalities. The country is divided into 47 prefectures (Fig. 1), with Hokkaido as the northernmost prefecture and Okinawa as the southernmost prefecture. Prefectures in Japan are medium-sized regions, resembling states in the United States, with only administrative power and not legislative power. There are 15 prefectures that have an ordinance-designated large city with a population > 500,000, including Hokkaido, Miyagi, Saitama, Tokyo, Chiba, Kanagawa, Niigata, Shizuoka, Aichi, Kyoto, Osaka, Hyogo, Okayama, Hiroshima, and Fukuoka. Referring to previous reports [22, 24], we investigated and compared two cases of urban–rural regional differences. (1) We defined the 15 prefectures as the large-city (urban) group and the other 32 as the non-large city (rural) group. (2) The top seven prefectures with a high population density > 1,000 persons per km^2^ were defined as the densely populated (urban) group and the remaining 40 prefectures as the sparsely populated (rural) group. The top seven most populated prefectures, namely Saitama, Tokyo, Chiba, Kanagawa, Aichi, Osaka, and Fukuoka, are located in the metropolitan areas and account for 45.7% of the total population in Japan.

### Statistical analysis

Correlations were assessed using the Pearson correlation method. Data comparisons between urban and rural groups were performed using the unpaired t-test. A two-sided *p*-value < 0.05 was considered statistically significant. All statistical analyses were performed using EZR (Saitama Medical Centre, Jichi Medical University, Saitama, Japan) [25], a graphical user interface for R (The R Foundation for Statistical Computing, Vienna, Austria).

## Results

Table 1 presents the annual nationwide incident subjects, number/incidence per 100,000 inhabitant/person-years and Gini coefficients for healthcare resources and representative surgical procedures during 2015–2019 for 47 prefectures. The Gini coefficients were low (< 0.2) in all, but moderate of 0.21 in the hospitals. Table 2 shows the relationships among representative surgical procedures, healthcare resources and the ageing rate. Femur fracture surgery and total knee arthroplasty were selected as the representative surgical procedures because in the general Japanese population, the femur fracture surgery had the highest incidence among the five types of fracture surgeries, and total knee arthroplasty had the higher incidence among arthroplasty surgery types (Table 1). Table 3 shows the results of the comparison between the two groups for regional differences in urban and rural prefectures. Supplementary Tables 1–7 show the individual data for each prefecture.

**Table 1:** The annual nationwide incident subjects, number/incidence per 100,000 inhabitant/person-years and Gini coefficients for healthcare resources and representative surgical procedures during 2015–2019, using 47 prefectures as the unit of analysis

				Annual nationwide incident subjects	Number/Incidence per 100,000 inhabitant/person-years			Gini coefficient
					Average (95% CI)	Maximum	Minimum	
Healthcare resource	Medical specialist	Orthopaedic surgeon specialist		-	14.2 (13.4 to 15.0)	19.0 (Kochi)	8.4 (Saitama)	0.11
		General surgeon specialist		-	17.6 (16.8 to 18.4)	25.4 (Kyoto)	11.9 (Niigata)	0.09
		Anaesthesiology specialist		-	5.9 (5.5 to 6.3)	8.5 (Shimane)	3.4 (Mie)	0.13
	Medical facility	Hospitals		-	8.1 (7.1 to 9.0)	18.1 (Kochi)	3.7 (Kanagawa)	0.21
		Beds^a^		-	1,411 (1307 to 1515)^a^	2545 (Kochi)^a^	806 (Kanagawa)^a^	0.14
Surgical procedure		Fracture surgery	Femur fracture surgery	121,934	110.6 (104.2 to 116.9)	149.2 (Tottori)	69.4 (Tokyo)	0.11
			Femoral head replacement	63,648	53.6 (50.2 to 57.0)	79.6 (Wakayama)	32.6 (Aomori)	0.13
			Forearm fracture surgery	56,584	47.1 (43.9 to 50.4)	74.2 (Oita)	23.6 (Miyagi)	0.14
			Lower leg fracture surgery	41,586	34.8 (33.3 to 36.3)	46.7 (Nagasaki)	26.4 (Miyagi)	0.09
			Upper arm fracture surgery	26,431	21.2 (20.0 to 22.4)	31.4 (Oita)	14.2 (Aichi)	0.12
		Arthroplasty surgery	Total knee arthroplasty	83,711	73.2 (67.4 to 79.0)	108.7 (Ehime)	32.3 (Miyagi)	0.16
			Total hip arthroplasty	58,870	47.2 (44.5 to 49.9)	76.4 (Saga)	25.3 (Iwate)	0.11
		General surgery	Cholecystectomy	12,4791	106.3 (101.2 to 111.4)	158.7 (Oita)	69.0 (Niigata)	0.09
			Appendectomy	55,478	44.0 (42.6 to 46.2)	66.4 (Okinawa)	31.4 (Aomori)	0.08

The Gini coefficient varies between 0 (complete equity) and 1 (complete inequity), according to the degree of variation in the number/incidence among the 47 prefectures. *CI* confidence interval

^a^The number of beds was only examined per 1,000 inhabitants

**Table 2:** Correlation coefficient of the number/incidence between representative surgeries, medical specialists, facilities, and the ageing rate

	Femur fracture surgery	Total knee arthroplasty	Cholecystectomy	Appendectomy	Orthopaedic surgeon specialist	General surgeon specialist	Anaesthesiology specialist	Hospitals	Ageing rate
Femur fracture surgery	1	**0.61**	**0.51**	0.23	**0.74**	**0.40**	**0.45**	**0.63**	**0.62**
Total knee arthroplasty		1	**0.55**	0.28	**0.64**	**0.36**	**0.52**	**0.61**	**0.42**
Cholecystectomy			1	**0.37**	**0.59**	**0.60**	**0.52**	**0.59**	**0.32**
Appendectomy				1	0.24	**0.37**	**0.45**	0.10	**-0.31**
Orthopaedic surgeon specialist					1	**0.69**	**0.67**	**0.67**	**0.43**
General surgeon specialist						1	**0.74**	**0.48**	0.15
Anaesthesiology specialist							1	**0.59**	0.08
Hospital								1	**0.54**
Ageing rate									1

Bold values indicate a statistically significant difference (*p* < 0.05)

**Table 3:** Regional analyses by the two urban/rural definitions for the number/incidence of representative surgeries, medical specialists, facilities, and the ageing rate

Urban/Rural		Fracture surgery					Arthroplasty surgery		General surgery		Medical specialist			Medical facility	Ageing rate
		Femur fracture surgery	Femoral head replacement	Forearm fracture surgery	Lower leg fracture surgery	Upper arm fracture surgery	Total knee arthroplasty	Total hip arthroplasty	Cholecyst-ectomy	Appendec-tomy	Orthopaedic surgeon specialist	General surgeon specialist	Anaesthes-iology specialist	Hospitals	
(1)	Large city (*n* = 15)	92.5 (17.3)	49.3 (8.4)	42.4 (10.0)	31.5 (3.9)	20.9 (4.8)	63.0 (18.0)	46.7 (8.8)	96.2 (18.0)	43.4 (4.7)	13.1 (2.5)	17.7 (3.9)	5.8 (1.5)	6.3 (2.0)	0.274 (0.023)
	Non-large city (*n* = 32)	119.0 (19.0)	55.6 (12.9)	49.3 (11.4)	36.3 (5.3)	21.3 (4.2)	77.9 (19.8)	47.4 (9.9)	111.1 (15.8)	44.9 (7.0)	14.7 (2.7)	17.6 (2.1)	5.9 (1.3)	8.9 (3.4)	0.304 (0.026)
	*p*-value	** < 0.001**	0.1	0.1	** < 0.01**	0.8	** < 0.05**	0.8	** < 0.01**	0.5	0.1	0.9	0.8	** < 0.01**	** < 0.001**
(2)	Densely populated area (*n* = 7)	81.8 (14.2)	47.1 (6.0)	43.8 (10.7)	31.2 (3.5)	20.7 (5.0)	56.9 (16.2)	44.7 (8.8)	90.0 (17.1)	43.4 (4.3)	12.4 (2.9)	16.9 (3.8)	5.6 (1.4)	5.3 (1.8)	0.256 (0.016)
	Sparsely populated area (*n* = 40)	115.6 (19.3)	54.7 (12.4)	47.7 (11.5)	35.4 (5.4)	21.3 (4.3)	76.0 (19.8)	47.6 (9.6)	109.2 (16.5)	44.6 (6.7)	14.5 (2.6)	17.7 (2.6)	6.0 (1.4)	8.5 (3.2)	0.302 (0.025)
	*p*-value	** < 0.001**	0.1	0.4	** < 0.05**	0.7	** < 0.05**	0.4	** < 0.01**	0.7	** < 0.05**	0.5	0.5	** < 0.05**	** < 0.001**

The number/incidence is reported per 100,000 inhabitant/person-years, except for the ageing rate. The results are expressed as mean (standard deviation). Bold *p*-values indicate a statistically significant difference (*p* < 0.05)

The national average ageing rate was 0.295, with the Akita Prefecture having the highest rate of 0.356 and Okinawa Prefecture having the lowest rate of 0.210.

**Fig. 2:**
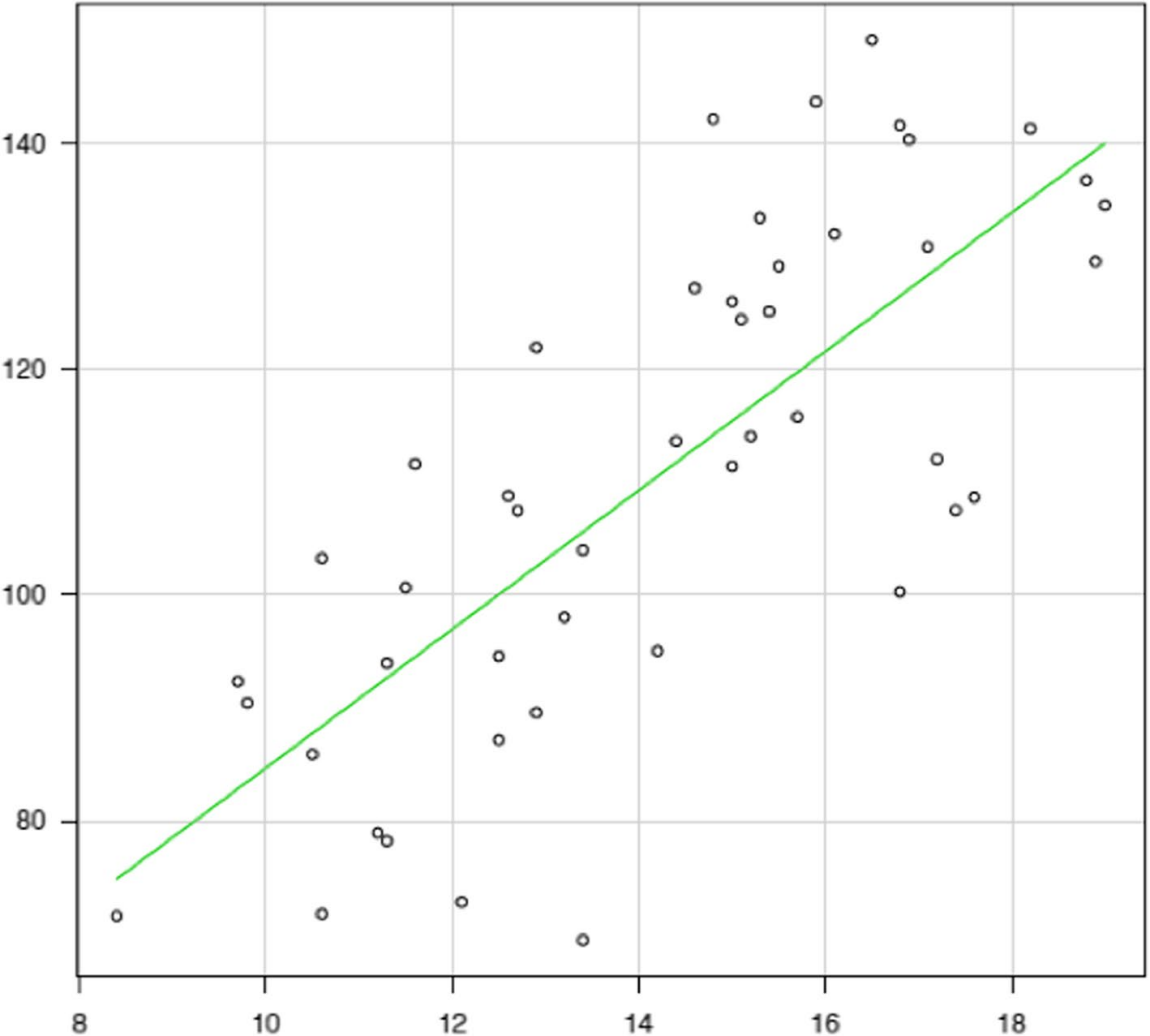
Correlation diagram of the number/incidence between orthopaedic surgeon specialist and Femur fracture surgery by prefecture The green line indicates the regression line (*R*^2^ = 0.546, *p* < 0.001)

**Fig. 3:**
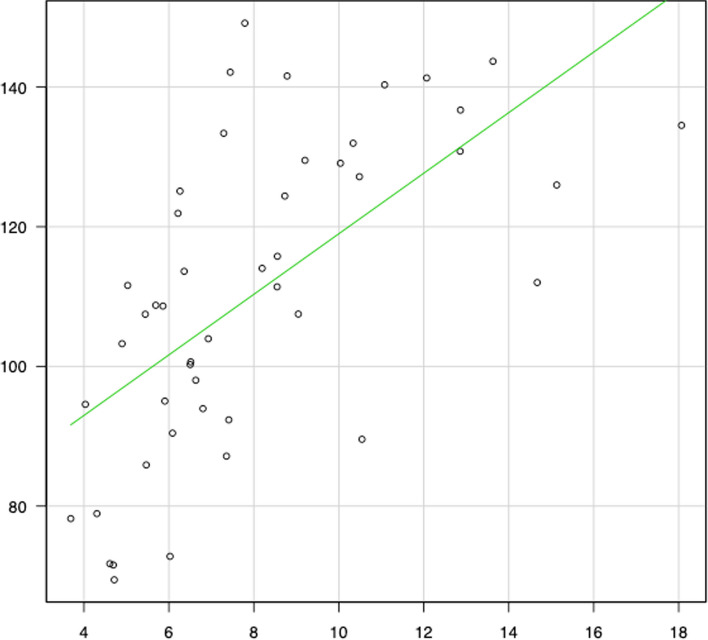
Correlation diagram of the number/incidence between hospitals and femur fracture surgery by prefecture The green line indicates the regression line (*R*^2^ = 0.401, *p* < 0.001)

**Fig. 4:**
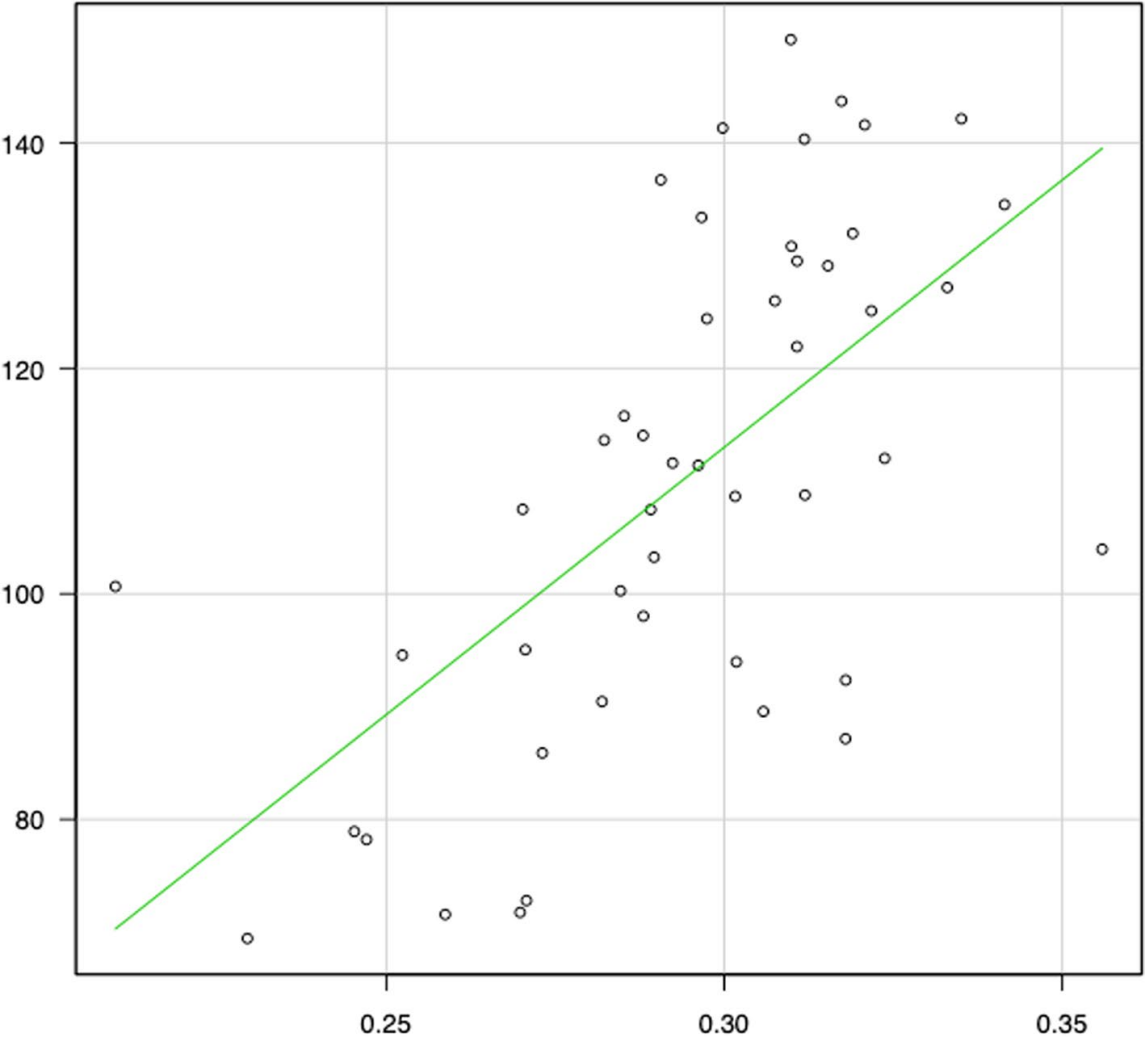
Correlation diagram of the number/incidence between the ageing rate and femur fracture surgery by prefecture The green line indicates the regression line (*R*^2^ = 0.384, *p* < 0.001)

**Fig. 5:**
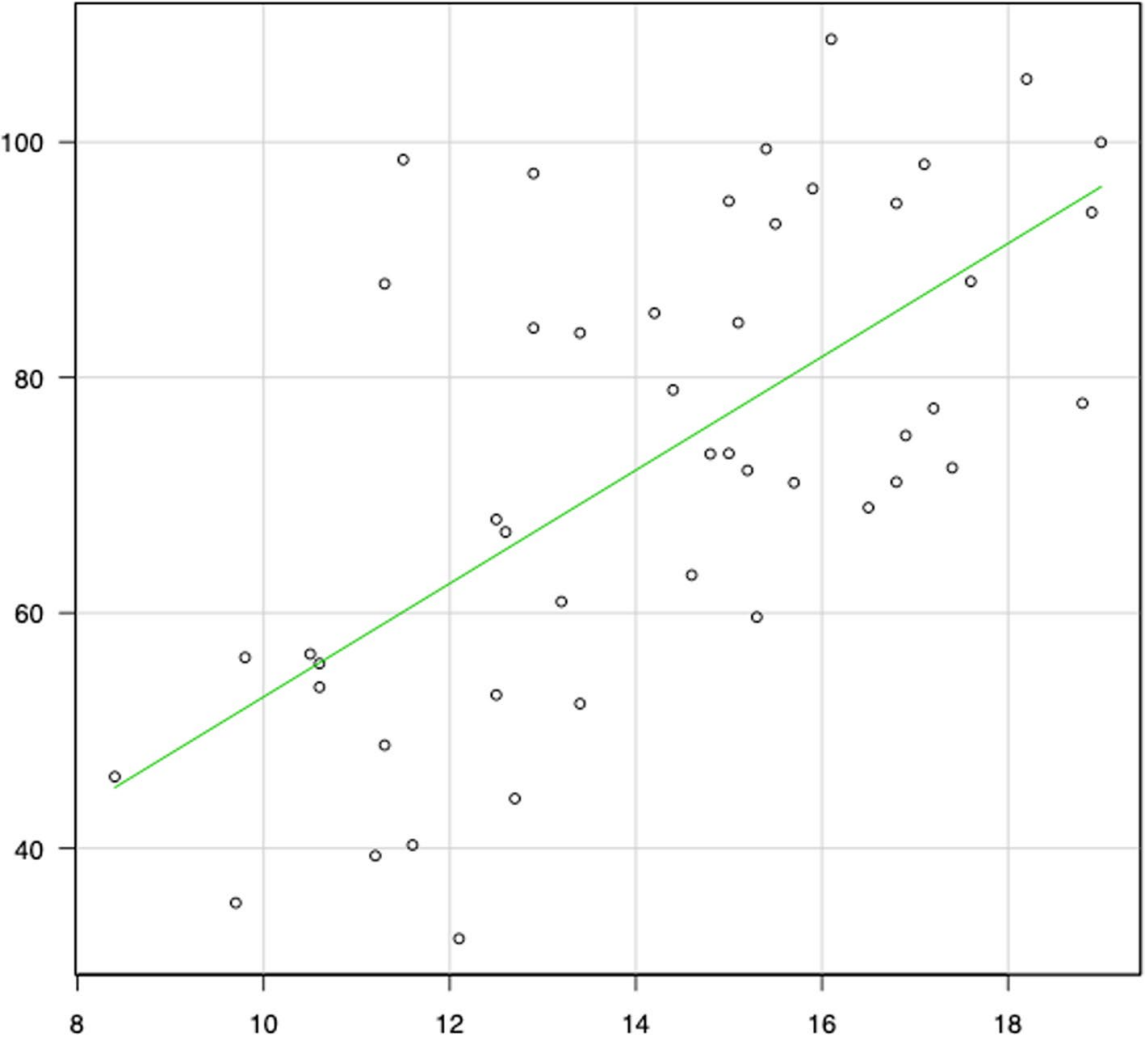
Correlation diagram of the number/incidence between orthopaedic surgeon specialist and total knee arthroplasty by prefecture The green line indicates the regression line (*R*^2^ = 0.405, *p* < 0.001)

**Fig. 6:**
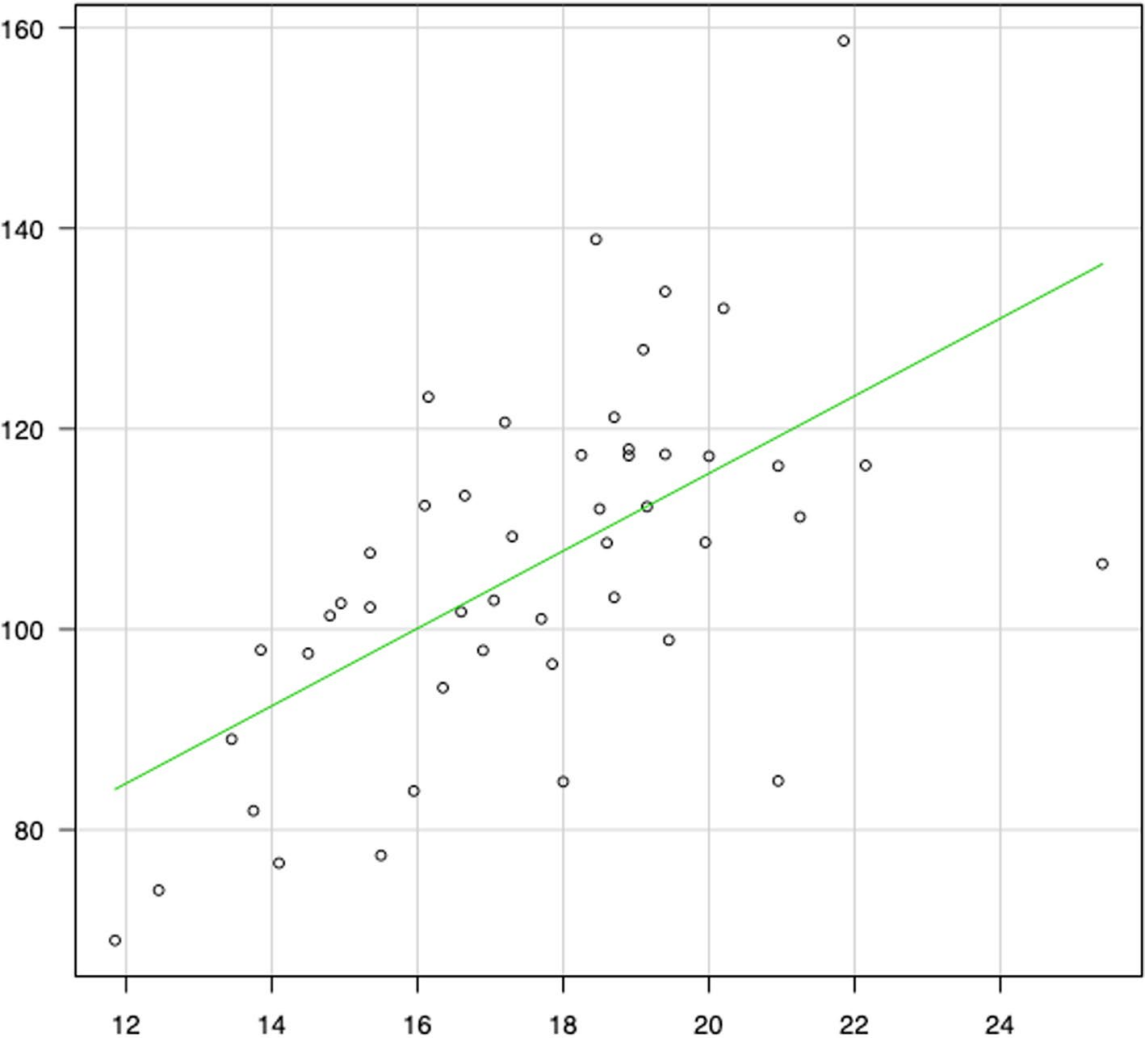
Correlation diagram of the number/incidence between general surgeon specialist and cholecystectomy by prefecture The green line indicates the regression line (*R*^2^ = 0.356, *p* < 0.001)

**Fig. 7:**
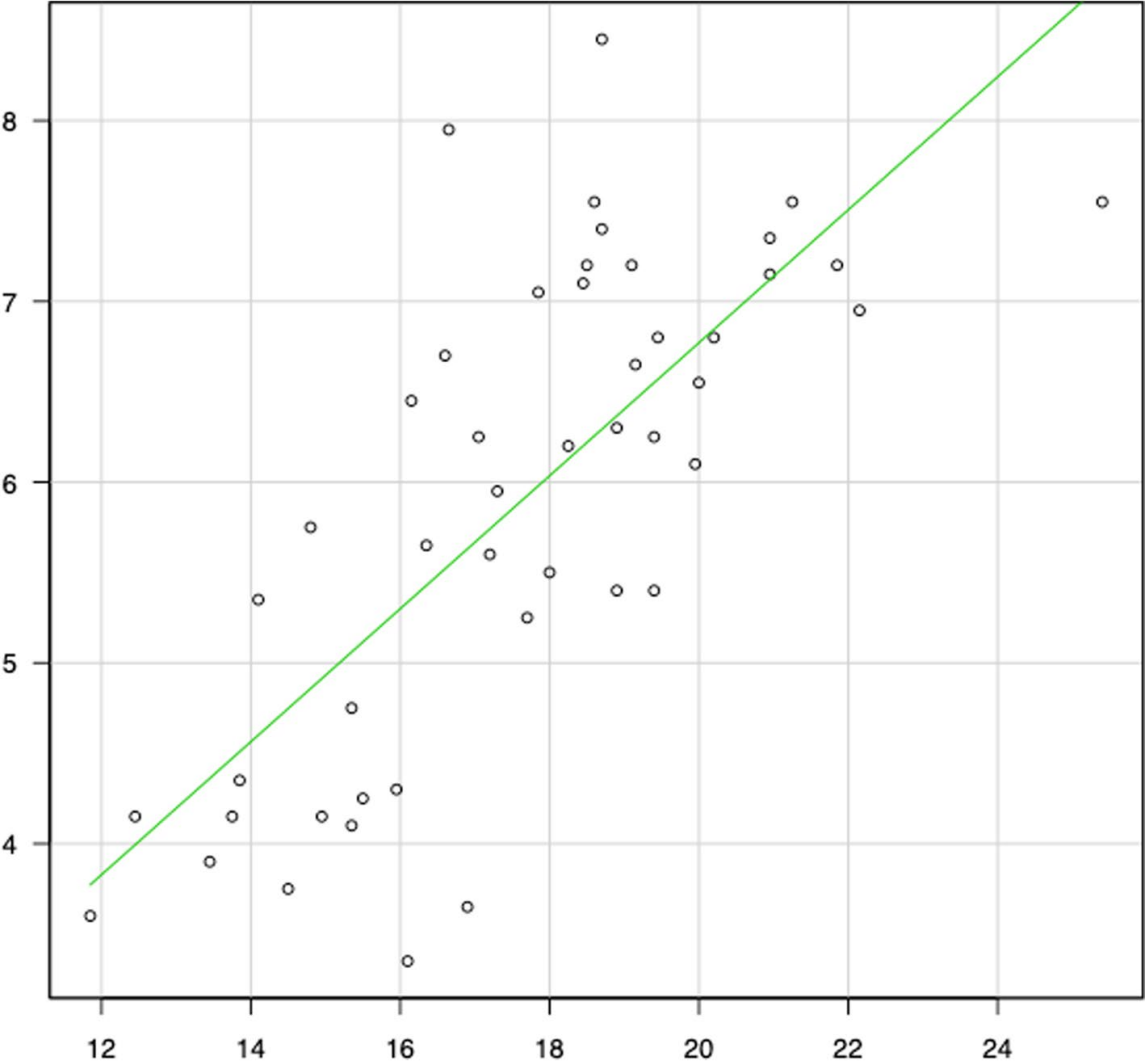
Correlation diagram of the number between general surgeon specialist and anaesthesiology specialist by prefecture The green line indicates the regression line (*R*^2^ = 0.542, *p* < 0.001)

Meanwhile, correlation diagrams are shown in Figs. 2, 3, 4, 5, 6 and 7. The correlation coefficients by the incidence of femur fracture surgery were 0.74 for orthopaedic surgeon specialists (*p* < 0.001) (Fig. 2), 0.63 for hospitals (*p* < 0.001) (Fig. 3), and 0.62 for the ageing rate (*p* < 0.001) (Fig. 4); those by the incidence of total knee arthroplasty was 0.64 for orthopaedic surgeon specialists (*p* < 0.001) (Fig. 5). The correlation coefficients by the incidence of cholecystectomy were 0.60 for general surgeon specialists (*p* < 0.001) (Fig. 6), 0.59 for hospitals (*p* < 0.001), and 0.32 for the ageing rate. The correlation coefficient between general surgeon specialists and anaesthesiology specialists was 0.74 (*p* < 0.001) (Fig. 7).

The regional differences between urban and rural areas were as follows. (1) The number/incidence of femur fracture surgery, lower leg fracture surgery, total knee arthroplasty, cholecystectomy, and hospitals, as well as the ageing rate, were significantly higher in the non-large city than in the large city. (2) The number/incidence of femur fracture surgery, lower leg fracture surgery, total knee arthroplasty, cholecystectomy, orthopaedic surgeon specialists, hospitals and the ageing rate were significantly higher in the sparsely populated area than in the densely populated area.

## Discussion

Using a nationwide database in Japan, we clarified regional disparities and relationships between healthcare resources and representative surgical procedures in orthopaedics and general surgery across 47 prefectures. Three factors (medical specialists, facilities, and representative surgical procedures) were found to correlate with each other. The inter-prefectural regional disparity was small, although certain items were unevenly distributed in the rural prefectures, which is contrary to our expectations.

The Gini coefficient indicated inequality and showed similar trends. The Gini coefficient was approximately 0.1 for all three medical specialists (Table 1), indicating that the regional disparity was small and homogeneous healthcare was provided at the prefectural level. Hence, the universal health insurance system in Japan [3] was effective at the prefectural level. Among the three types of specialists, the general surgeon specialists had the smallest Gini coefficient, followed by the orthopaedic surgeon specialists and anaesthesiology specialist, which increased slightly. This difference may result from the effect of the parameter size. Meaningful maldistribution of physicians was not confirmed at the prefectural level, although it has been reported at the municipal level previously [6]. It has also been reported that the inter-prefectural disparity in the incidence of paediatricians was smaller than the inter-municipal (intra-prefectural) disparity [22], which should be interpreted with caution. However, there have been no reports of regional disparities in the incidence of surgical procedures using the Gini coefficient. Among surgical procedures in orthopaedics, the Gini coefficient was the lowest (0.09) for lower limb fracture surgery. When comparing representative surgical procedures between orthopaedics and general surgery, those in general surgery had slightly lower Gini coefficients (0.08 and 0.09, respectively). This reflects the necessity or urgency of the lower limb fracture surgery and the two general surgeries (cholecystectomy and appendectomy). The Gini coefficient of medical facilities was higher for hospitals than for beds. This was also considered to be due to the effect of the parameter size. Regardless, all Gini coefficients were approximately 0.1–0.2 (Table 1), indicating that the regional differences were small at the prefectural level.

Correlation analysis revealed strong to moderate positive correlations between the number/incidence of certain surgical procedures, orthopaedic surgeon specialists, general surgeon specialists, and hospitals (Table 2, Figs. 2, 3, 4, 5, 6 and 7). The incidences of femur fracture surgery and total knee arthroplasty showed strong to moderate positive correlations with the ageing rate, orthopaedic surgeon specialists, and hospitals, while the cholecystectomy incidence exhibited a moderate positive correlation with general surgeon specialists and hospitals. The appendectomy incidence showed a moderate positive correlation with general surgeon specialists and a moderate negative correlation with the ageing rate. This may reflect surgical characteristics such as urgency, necessity, and the age structure of the disease (Supplementary Figures 1, 2, 4, and 5). Additionally, medical specialists and facilities correlated with each other, reflecting a symbiotic relationship. The incidence of surgical procedures is determined by patient demand and medical/healthcare supply. On the patient side, the demand for surgery may be influenced by disease prevalence, the nature of the disease (urgency), the patient’s knowledge of the disease (literacy), and their willingness to undergo surgery. Meanwhile, the supply on the medical side may depend on the number of physicians and facilities and the quality of both (surgeon’s criteria for surgery and ability to diagnose and perform surgery). Accordingly, in this study, we found that the number/incidence of medical specialists, facilities, and surgical procedures were correlated, as expected.

However, unexpectedly, the number/incidence of certain surgical procedures in orthopaedics and general surgery, orthopaedic surgeon specialists (only in sparsely populated areas), and the ageing rate were all higher in rural prefectures than in urban prefectures (Table 3). Investigating the age-stratified data in 2019 for femur fracture surgery, total knee arthroplasty, lower leg fracture surgery, and cholecystectomy revealed that a peak was centred on the elderly in all cases (Supplementary Figures 1–4). However, the distributions differ significantly, suggesting that the population ageing affected other parameters. Although it was biased toward rural prefectures, we considered that the supply–demand balance was maintained among the three parties (medical specialists, facilities, and surgical procedures).

Few studies have examined localized regional disparities in healthcare using the incidence of surgical procedures. A Swedish study [26] of orthopaedic foot surgery speculated that the concentration of specialists increased the incidence of surgery in urban areas. Evidently, the data documented in Japan provided a different spectrum in which the incidence in urban prefectures was slightly lower than that in rural prefectures. However, it is difficult to make simple direct comparisons due to differences in the definitions of urban–rural, race, culture, social structure, and other factors across countries.

Collectively, the present study’s results indicate that, at the prefectural level in Japan, the supply–demand balance in medical specialists, facilities, and representative surgeries has been maintained and that there is little disparity among medium-sized regions. Sufficient healthcare is guaranteed in rural areas, even with the growing ageing population. The Japanese medical system for typical surgical treatments in orthopaedics and general surgery was successful from 2015 to 2019. Accordingly, the results of this study provide important insights to inform healthcare policy planning. Additional validation is needed to follow the longitudinal data in the future.

The OECD [27] publishes annual data on representative surgical procedures, including total knee arthroplasty, hip replacement, appendectomy, and cholecystectomy. Accordingly, we compared the surgical incidence per 100,000 people reported in OECD countries in North America, Europe, and East Asia between 2015 and 2019 with our results (Table 4). In Japan, the incidence of the four surgeries was lower than in other countries, except for hip replacement in South Korea. However, these data cannot be directly compared due to differences in race, ethnicity, and social systems, including healthcare, as well as subtle differences in surgical codes indicating the slightly different definitions of surgeries. Nevertheless, this study of regional epidemiology is intended to provide important information for international medical societies.

**Table 4:** Multinational comparison of the annual incidence of representative surgical procedures in orthopaedics and general surgery during 2015–2019 (per 100,000 people)

		2015	2016	2017	2018	2019
Total knee arthroplasty	Canada	178.6	184.9	191.6	201.9	198.3
	France	160.1	167.1	174.3	181.3	185
	Germany	205.8	218.6	223.4	222.8	227.4
	Sweden	123.7	117.4	138.6	130.6	134.6
	Korea	121 (B)	136.2	135.8	139.1	153.3
	Japan	60.6	60.6	65.0	69.6	74.7
Hip replacement	Canada	148.4	154.3	159.4	166.5	168.1
	France	240.8	242.9	247.8	247.9	251.5
	Germany	299.3	304.4	309.4	310.6	314.9
	Sweden	234.2	226	245.7	242	242.8
	Korea	53.4	54.4	55.9	56.8	58.8
	Japan	88.1	89.8	97.9	101.9	106.0
Appendectomy	Canada	107.4	107.9	109.1	112.3	111.1
	France	115.3	108.6	106.9	107.2	106.2
	Germany	159.6	155.1	152.8	149.5	144.1
	Sweden	133.3	130.1	127.7	127	124.7
	Korea	177.4	181.3	176.3	160	157.6
	Japan	44.9	43.2	43.1	43.5	44.3
Cholecystectomy	Canada	207.4	205.7	204.5	206.3	198.1
	France	196.1	197.9	195	192.7	189.9
	Germany	244.5	247.8	242.5	239.8	244.8
	Sweden	143.3	142.4	138.9	137.7	144.1
	Korea	124	136.2	142.7	152.2	163.2
	Japan	96.6	97.1	98.7	100.0	100.1

This table was created based on data from our study and the OECD statistics ( https://stats.oecd.org/Index.aspx?DatasetCode=HEALTH_STAT, accessed 3 Nov 2022)

(*B*) break, *OECD *Organization for Economic Co-operation and Development

This study has several limitations. (1) The study did not focus on disease prevalence but on surgical procedures. (2) The number of medical specialists was surveyed using a biennial questionnaire. However, the estimated registry rate was reported to be 87–90% [28]. (3) The survey on the number of medical facilities was conducted every three years, and the results for only 2017 were used. (4) As aforementioned, the survey methods of surgical procedures are not standardised and may differ slightly from country to country; this issue has also been previously reported [15].

In conclusion, this is the first observational epidemiological study on regional disparities and relationships between healthcare resources and representative surgical procedures in orthopaedics and general surgery. Inter-prefectural regional disparity was small, although certain items were unevenly distributed in the rural prefectures. Higher prevalence was recognised in rural prefectures than urban prefectures due to the higher ageing rate; however, supply and demand were relatively balanced. This study provides basic data for healthcare policy development in a medium-sized community.

### Supplementary Information

**Additional file 1: Supplementary Figure 1.** Age-stratified incidence of femur fracture surgery in 2019. **Supplementary Figure 2.** Age-stratified incidence of knee arthroplasty in 2019.** Supplementary Figure 3.** Age-stratified incidence of lower leg fracture surgery in 2019. **Supplementary Figure 4.** Age-stratified incidence of cholecystectomy in 2019. **Supplementary Figure 5.** Age-stratified incidence of appendectomy in 2019. **Supplementary Table 1.** Prefectural incidence of femur fracture surgery during 2015–2019 (per 100,000 people). **Supplementary Table 2.** Prefectural incidence of knee arthroplasty during 2015–2019 (per 100,000 people). **Supplementary Table 3.** Prefectural incidence of cholecystectomy during 2015–2019 (per 100,000 people). **Supplementary Table 4.** Prefectural incidence of appendectomy during 2015–2019 (per 100,000 people). **Supplementary Table 5.** Prefectural number of medical specialists in orthopaedics, general surgery, and anaesthesiology during 2015–2019 (per 100,000 inhabitants). **Supplementary Table 6.** Prefectural number of medical facilities (hospitals and beds) during 2015–2019 (per 100,000 inhabitants for hospitals and per 1,000 inhabitants for beds). **Supplementary Table 7.** Prefectural index of the ageing rate during 2015–2019

## Data Availability

The datasets used during the current study are available from the following public domain resources: https://www.mhlw.go.jp/stf/seisakunitsuite/bunya/0000177182.html https://www.e-stat.go.jp/en/stat-search/ https://stats.oecd.org/Index.aspx?DatasetCode=HEALTH_STAT.
