# Supplementary material for: The inter-prefectural regional disparity of healthcare resources and representative surgical procedures in orthopaedics and general surgery: a nationwide study in Japan during 2015–2019
Source: BMC Musculoskelet Disord. 2023 Sep 12;24:726. doi: 10.1186/s12891-023-06820-0 (PMC10496376; doi:10.1186/s12891-023-06820-0)
Supplement: Supplementary file 1 — Additional file 1: Supplementary Figure 1. Age-stratified incidence of femur fracture surgery in 2019. Supplementary Figure 2. Age-stratified incidence of knee arthroplasty in 2019. Supplementary Figure 3. Age-stratified incidence of lower leg fracture surgery in 2019. Supplementary Figure 4. Age-stratified incidence of cholecystectomy in 2019. Supplementary Figure 5. Age-stratified incidence of appendectomy in 2019. Supplementary Table 1. Prefectural incidence of femur fracture surgery during 2015–2019 (per 100,000 people). Supplementary Table 2. Prefectural incidence of knee arthroplasty during 2015–2019 (per 100,000 people). Supplementary Table 3. Prefectural incidence of cholecystectomy during 2015–2019 (per 100,000 people). Supplementary Table 4. Prefectural incidence of appendectomy during 2015–2019 (per 100,000 people). Supplementary Table 5. Prefectural number of medical specialists in orthopaedics, general surgery, and anaesthesiology during 2015–2019 (per 100,000 inhabitants). Supplementary Table 6. Prefectural number of medical facilities (hospitals and beds) during 2015–2019 (per 100,000 inhabitants for hospitals and per 1,000 inhabitants for beds). Supplementary Table 7. Prefectural index of the ageing rate during 2015–2019 [file 12891_2023_6820_MOESM1_ESM.docx]

**
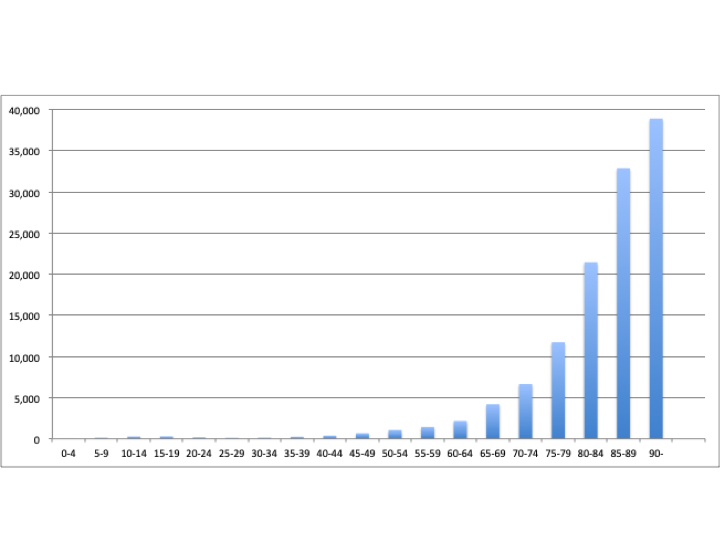
Supplementary Figure 1** Age-stratified incidence of femur fracture surgery in 2019.

A unimodal distribution is observed with a peak at >90 years. All supplementary figures are created based on data from the NDB Open Data Japan^19^ and the demographics of the Ministry of Internal Affairs and Communications^20^.

**
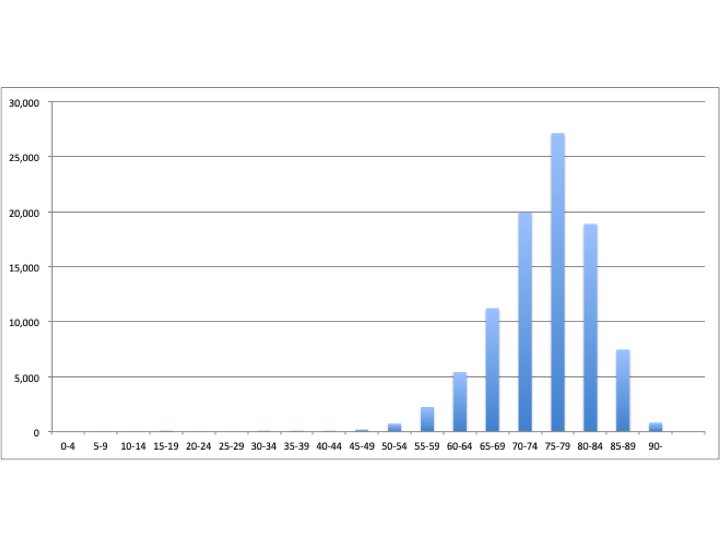
**

**Supplementary Figure 2.** Age-stratified incidence of knee arthroplasty in 2019.

A unimodal distribution is observed with a peak at 75–79 years.


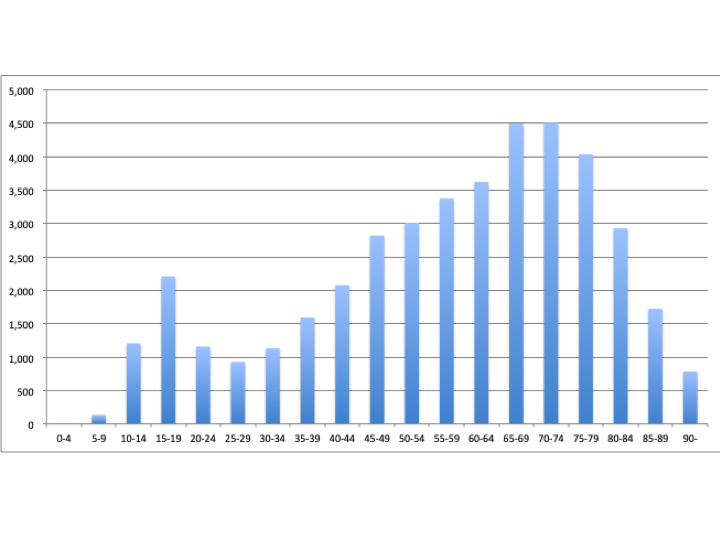
**Supplementary Figure 3.** Age-stratified incidence of lower leg fracture surgery in 2019.

A bimodal distribution is observed with peaks at 15–19 and 65–74 years.


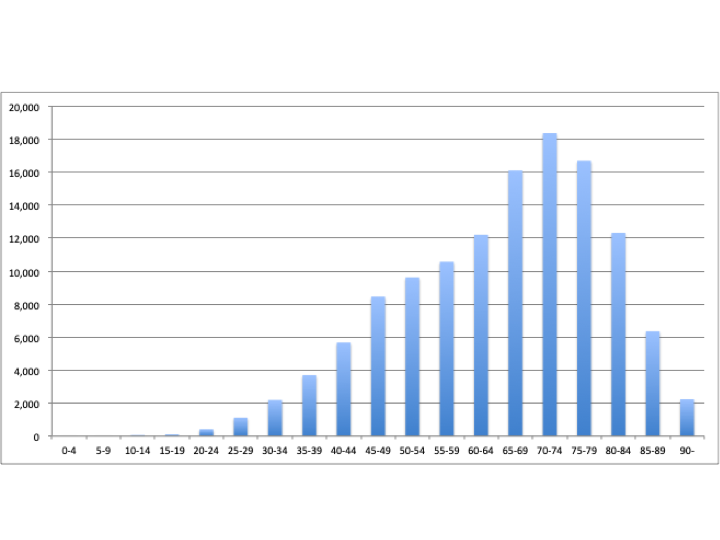


**Supplementary Figure 4.** Age-stratified incidence of cholecystectomy in 2019.

A unimodal distribution is observed with a peak at 70–74 years.

**
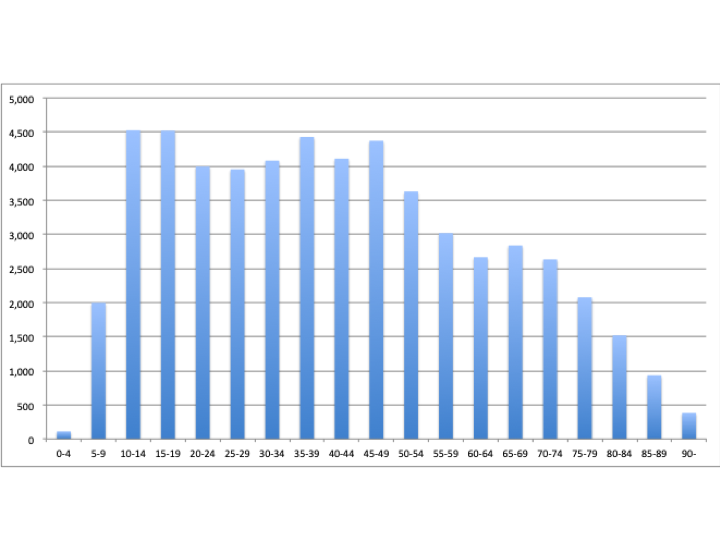
**

**Supplementary Figure 5.** Age-stratified incidence of appendectomy in 2019.

An approximately bimodal distribution is observed with peaks at 10–19 and 35–49 years.

Supplementary Table 1 Prefectural incidence of femur fracture surgery during 2015–2019 (per 100,000 people)

| Year | 2015 | 2016 | 2017 | 2018 | 2019 |
| --- | --- | --- | --- | --- | --- |
| National average | 93.5 | 95.0 | 98.3 | 97.0 | 97.5 |
| Name of the prefecture |  |  |  |  |  |
| Hokkaido | 84.7 | 87.4 | 91.8 | 92.1 | 92.0 |
| Aomori | 85.5 | 84.8 | 89.9 | 91.8 | 83.7 |
| Iwate | 90.0 | 91.6 | 94.7 | 93.4 | 92.2 |
| Miyagi | 68.4 | 72.7 | 73.6 | 74.7 | 74.8 |
| Akita | 100.3 | 97.0 | 106.5 | 107.6 | 108.7 |
| Yamagata | 122.3 | 121.0 | 128.9 | 129.5 | 123.7 |
| Fukushima | 89.9 | 90.6 | 98.5 | 92.5 | 98.4 |
| Ibaraki | 89.3 | 89.3 | 94.5 | 89.4 | 89.7 |
| Tochigi | 85.1 | 86.0 | 83.4 | 86.2 | 88.8 |
| Gumma | 100.9 | 93.8 | 102.2 | 93.7 | 99.4 |
| Saitama | 68.0 | 68.3 | 74.3 | 73.0 | 74.2 |
| Chiba | 68.7 | 69.5 | 72.3 | 73.0 | 75.3 |
| Tokyo | 67.5 | 67.5 | 72.4 | 69.6 | 70.1 |
| Kanagawa | 75.5 | 78.5 | 77.8 | 78.0 | 81.3 |
| Niigata | 101.9 | 107.8 | 112.7 | 108.5 | 113.1 |
| Toyama | 126.2 | 128.5 | 135.8 | 129.8 | 125.2 |
| Ishikawa | 108.8 | 107.3 | 119.5 | 116.5 | 118.1 |
| Fukui | 118.3 | 120.1 | 129.7 | 130.6 | 123.4 |
| Yamanashi | 129.6 | 131.9 | 137.3 | 133.3 | 134.9 |
| Nagano | 120.4 | 123.0 | 118.6 | 126.3 | 121.2 |
| Gifu | 111.3 | 114.2 | 111.3 | 109.6 | 111.6 |
| Shizuoka | 101.3 | 100.6 | 106.9 | 101.9 | 105.6 |
| Aichi | 76.0 | 79.8 | 80.3 | 78.6 | 79.9 |
| Mie | 105.7 | 108.9 | 108.6 | 107.2 | 107.0 |
| Shiga | 95.9 | 95.8 | 93.7 | 92.4 | 95.0 |
| Kyoto | 97.9 | 100.3 | 99.9 | 103.2 | 100.0 |
| Osaka | 92.3 | 93.2 | 95.9 | 97.0 | 96.7 |
| Hyogo | 108.6 | 111.2 | 116.3 | 116.3 | 115.7 |
| Nara | 102.3 | 107.4 | 109.1 | 111.0 | 113.6 |
| Wakayama | 132.8 | 133.8 | 145.1 | 147.1 | 149.7 |
| Tottori | 144.5 | 143.5 | 153.3 | 155.2 | 149.5 |
| Shimane | 134.4 | 140.9 | 157.1 | 141.0 | 137.2 |
| Okayama | 116.8 | 112.6 | 115.0 | 106.3 | 106.2 |
| Hiroshima | 110.9 | 110.2 | 116.7 | 122.3 | 118.8 |
| Yamaguchi | 129.3 | 131.6 | 127.7 | 122.0 | 125.1 |
| Tokushima | 106.6 | 115.2 | 111.6 | 109.6 | 117.2 |
| Kagawa | 121.7 | 127.2 | 137.7 | 134.3 | 126.7 |
| Ehime | 126.8 | 130.4 | 132.2 | 128.8 | 141.8 |
| Kochi | 137.8 | 132.5 | 140.5 | 134.7 | 126.9 |
| Fukuoka | 104.7 | 105.8 | 112.4 | 106.5 | 108.1 |
| Saga | 128.6 | 136.4 | 135.6 | 145.4 | 137.8 |
| Nagasaki | 136.7 | 138.0 | 146.8 | 140.6 | 139.6 |
| Kumamoto | 134.7 | 148.8 | 140.0 | 141.2 | 141.8 |
| Oita | 136.7 | 146.4 | 147.7 | 144.8 | 142.8 |
| Miyazaki | 130.8 | 129.2 | 127.2 | 130.1 | 136.9 |
| Kagoshima | 123.6 | 121.9 | 130.9 | 127.6 | 126.0 |
| Okinawa | 98.9 | 97.8 | 101.5 | 104.8 | 100.3 |

Supplementary Table 2 Prefectural incidence of knee arthroplasty during 2015–2019 (per 100,000 people)

| Year | 2015 | 2016 | 2017 | 2018 | 2019 |
| --- | --- | --- | --- | --- | --- |
| National average | 60.6 | 60.6 | 65.0 | 69.6 | 74.7 |
| Name of the prefecture |  |  |  |  |  |
| Hokkaido | 90.1 | 89.9 | 98.3 | 104.2 | 104.5 |
| Aomori | 59.2 | 62.5 | 70.1 | 71.8 | 76.6 |
| Iwate | 37.0 | 36.2 | 32.2 | 36.5 | 34.9 |
| Miyagi | 32.2 | 29.3 | 30.5 | 32.9 | 36.7 |
| Akita | 92.5 | 74.5 | 77.7 | 85.5 | 88.8 |
| Yamagata | 105.5 | 95.7 | 97.0 | 96.5 | 102.3 |
| Fukushima | 85.1 | 84.2 | 84.0 | 94.9 | 91.9 |
| Ibaraki | 47.1 | 49.8 | 57.6 | 62.4 | 64.3 |
| Tochigi | 55.3 | 53.4 | 55.3 | 59.3 | 59.4 |
| Gumma | 50.3 | 54.3 | 59.0 | 66.2 | 75.2 |
| Saitama | 39.7 | 40.2 | 45.9 | 49.7 | 54.9 |
| Chiba | 48.9 | 49.2 | 52.9 | 56.8 | 60.7 |
| Tokyo | 48.6 | 47.7 | 51.4 | 55.2 | 58.4 |
| Kanagawa | 43.8 | 44.0 | 49.4 | 52.7 | 53.9 |
| Niigata | 65.3 | 60.8 | 66.4 | 69.5 | 72.6 |
| Toyama | 82.1 | 87.7 | 91.0 | 98.6 | 106.1 |
| Ishikawa | 68.5 | 68.0 | 67.0 | 72.7 | 84.4 |
| Fukui | 77.0 | 70.7 | 80.7 | 96.5 | 98.7 |
| Yamanashi | 60.1 | 53.9 | 65.6 | 59.4 | 59.3 |
| Nagano | 82.5 | 83.7 | 79.3 | 87.3 | 88.3 |
| Gifu | 37.0 | 37.6 | 39.9 | 42.1 | 44.9 |
| Shizuoka | 48.3 | 51.5 | 52.0 | 59.0 | 68.0 |
| Aichi | 35.5 | 37.9 | 37.5 | 40.6 | 45.4 |
| Mie | 43.1 | 39.0 | 41.7 | 44.7 | 52.8 |
| Shiga | 46.7 | 49.0 | 50.7 | 56.0 | 62.7 |
| Kyoto | 66.2 | 67.5 | 68.2 | 69.6 | 84.3 |
| Osaka | 73.8 | 78.2 | 83.0 | 91.5 | 100.9 |
| Hyogo | 73.7 | 74.8 | 79.9 | 80.5 | 85.9 |
| Nara | 81.5 | 74.6 | 84.1 | 94.0 | 107.1 |
| Wakayama | 80.6 | 89.2 | 102.9 | 97.3 | 104.5 |
| Tottori | 63.5 | 61.6 | 66.5 | 75.2 | 78.2 |
| Shimane | 75.1 | 75.1 | 73.1 | 73.4 | 70.8 |
| Okayama | 63.5 | 65.3 | 72.3 | 76.0 | 90.9 |
| Hiroshima | 68.7 | 64.8 | 68.6 | 73.6 | 79.7 |
| Yamaguchi | 58.0 | 57.5 | 56.3 | 66.4 | 78.2 |
| Tokushima | 76.5 | 71.2 | 79.7 | 76.9 | 82.8 |
| Kagawa | 79.5 | 87.8 | 98.3 | 98.0 | 106.8 |
| Ehime | 85.9 | 95.2 | 107.0 | 116.7 | 139.9 |
| Kochi | 96.4 | 94.3 | 95.1 | 104.8 | 109.6 |
| Fukuoka | 66.7 | 66.1 | 72.8 | 75.8 | 80.2 |
| Saga | 77.3 | 70.5 | 73.3 | 80.8 | 87.2 |
| Nagasaki | 71.1 | 69.4 | 70.7 | 78.4 | 86.1 |
| Kumamoto | 98.5 | 93.3 | 100.6 | 111.3 | 123.4 |
| Oita | 89.7 | 85.8 | 99.2 | 101.7 | 104.1 |
| Miyazaki | 87.2 | 85.9 | 101.2 | 106.8 | 110.0 |
| Kagoshima | 87.0 | 81.7 | 90.9 | 103.6 | 112.2 |
| Okinawa | 89.1 | 91.0 | 97.5 | 106.5 | 108.3 |

Supplementary Table 3 Prefectural incidence of cholecystectomy during 2015–2019 (per 100,000 people)

| Year | 2015 | 2016 | 2017 | 2018 | 2019 |
| --- | --- | --- | --- | --- | --- |
| National average | 96.6 | 97.1 | 98.7 | 100.0 | 100.1 |
| Name of the prefecture |  |  |  |  |  |
| Hokkaido | 112.5 | 111.9 | 115.7 | 114.1 | 112.5 |
| Aomori | 110.8 | 107.0 | 105.2 | 104.6 | 110.4 |
| Iwate | 103.1 | 97.6 | 93.4 | 99.0 | 94.6 |
| Miyagi | 115.8 | 112.8 | 118.3 | 122.9 | 117.4 |
| Akita | 97.6 | 97.9 | 94.1 | 104.6 | 95.3 |
| Yamagata | 99.1 | 105.2 | 103.1 | 101.5 | 104.2 |
| Fukushima | 101.3 | 102.1 | 101.7 | 102.0 | 104.0 |
| Ibaraki | 94.7 | 95.4 | 99.2 | 102.2 | 98.3 |
| Tochigi | 98.0 | 101.4 | 99.8 | 99.3 | 106.8 |
| Gumma | 99.9 | 101.6 | 103.9 | 103.9 | 105.1 |
| Saitama | 70.9 | 71.1 | 74.1 | 76.2 | 77.5 |
| Chiba | 79.7 | 81.4 | 85.7 | 87.1 | 85.3 |
| Tokyo | 84.9 | 84.7 | 85.4 | 83.6 | 85.8 |
| Kanagawa | 74.7 | 74.4 | 74.5 | 78.3 | 81.5 |
| Niigata | 68.2 | 67.6 | 69.6 | 69.5 | 70.0 |
| Toyama | 93.2 | 96.4 | 99.4 | 107.0 | 113.0 |
| Ishikawa | 100.3 | 94.6 | 97.9 | 101.9 | 99.9 |
| Fukui | 121.9 | 112.8 | 122.3 | 115.4 | 117.4 |
| Yamanashi | 86.6 | 89.0 | 89.9 | 81.2 | 77.1 |
| Nagano | 93.4 | 89.1 | 91.7 | 93.7 | 103.1 |
| Gifu | 90.5 | 87.3 | 87.7 | 89.7 | 89.9 |
| Shizuoka | 75.2 | 75.8 | 78.4 | 78.5 | 79.3 |
| Aichi | 79.8 | 81.7 | 81.7 | 84.9 | 81.4 |
| Mie | 106.4 | 112.5 | 110.2 | 118.1 | 114.7 |
| Shiga | 98.4 | 104.5 | 99.1 | 102.7 | 102.1 |
| Kyoto | 104.8 | 108.3 | 107.8 | 108.1 | 103.6 |
| Osaka | 109.2 | 110.7 | 112.8 | 113.9 | 114.6 |
| Hyogo | 106.9 | 110.4 | 109.3 | 109.0 | 110.6 |
| Nara | 115.1 | 116.2 | 120.8 | 124.6 | 126.7 |
| Wakayama | 117.7 | 118.9 | 120.0 | 111.3 | 118.6 |
| Tottori | 136.3 | 125.8 | 139.8 | 128.9 | 129.1 |
| Shimane | 96.0 | 95.9 | 110.8 | 98.4 | 115.1 |
| Okayama | 107.4 | 112.2 | 111.2 | 117.3 | 107.9 |
| Hiroshima | 108.4 | 107.5 | 106.4 | 110.8 | 110.3 |
| Yamaguchi | 121.1 | 113.6 | 113.2 | 123.1 | 115.9 |
| Tokushima | 124.6 | 138.1 | 137.6 | 146.6 | 148.1 |
| Kagawa | 124.8 | 124.3 | 133.3 | 124.3 | 132.8 |
| Ehime | 128.2 | 135.1 | 124.6 | 140.4 | 140.4 |
| Kochi | 110.3 | 109.7 | 123.4 | 123.2 | 115.0 |
| Fukuoka | 115.4 | 111.8 | 119.1 | 118.9 | 116.6 |
| Saga | 94.5 | 94.1 | 90.0 | 100.7 | 103.4 |
| Nagasaki | 112.7 | 119.5 | 111.2 | 121.6 | 121.4 |
| Kumamoto | 121.8 | 119.8 | 123.7 | 118.8 | 121.6 |
| Oita | 151.7 | 153.9 | 156.9 | 170.6 | 160.7 |
| Miyazaki | 105.2 | 100.4 | 113.0 | 112.0 | 112.7 |
| Kagoshima | 109.3 | 113.0 | 116.9 | 108.9 | 111.9 |
| Okinawa | 117.5 | 124.0 | 125.4 | 126.4 | 122.5 |

Supplementary Table 4 Prefectural incidence of appendectomy during 2015–2019 (per 100,000 people).

| Year | 2015 | 2016 | 2018 | 2019 |
| --- | --- | --- | --- | --- |
| National average | 44.9 | 43.2 | 43.5 | 44.3 |
| Name of Prefecture |  |  |  |  |
| Hokkaido | 46.8 | 43.6 | 47.3 | 44.0 |
| Aomori | 34.4 | 31.2 | 30.8 | 28.9 |
| Iwate | 46.5 | 44.1 | 38.8 | 40.4 |
| Miyagi | 45.4 | 42.4 | 41.8 | 40.7 |
| Akita | 44.0 | 38.0 | 43.6 | 39.6 |
| Yamagata | 43.6 | 36.5 | 38.2 | 36.6 |
| Fukushima | 41.9 | 40.8 | 37.1 | 38.0 |
| Ibaraki | 43.5 | 42.7 | 42.5 | 42.9 |
| Tochigi | 38.6 | 34.1 | 37.1 | 39.1 |
| Gumma | 39.2 | 35.0 | 38.5 | 38.8 |
| Saitama | 37.1 | 37.2 | 35.9 | 37.6 |
| Chiba | 39.2 | 38.8 | 40.6 | 40.0 |
| Tokyo | 47.3 | 46.8 | 46.1 | 48.0 |
| Kanagawa | 45.2 | 41.5 | 43.1 | 45.0 |
| Niigata | 36.0 | 34.3 | 33.6 | 31.2 |
| Toyama | 47.4 | 52.5 | 49.6 | 49.0 |
| Ishikawa | 57.6 | 57.9 | 56.4 | 55.4 |
| Fukui | 54.1 | 52.6 | 56.1 | 56.3 |
| Yamanashi | 37.8 | 39.0 | 39.5 | 36.0 |
| Nagano | 50.6 | 46.5 | 52.5 | 54.3 |
| Gifu | 46.9 | 43.8 | 42.4 | 42.8 |
| Shizuoka | 39.8 | 39.0 | 36.2 | 36.3 |
| Aichi | 43.3 | 41.7 | 39.5 | 41.9 |
| Mie | 46.5 | 47.2 | 42.4 | 44.6 |
| Shiga | 46.9 | 50.0 | 45.5 | 46.7 |
| Kyoto | 51.0 | 50.5 | 49.2 | 46.6 |
| Osaka | 46.6 | 44.0 | 46.4 | 48.1 |
| Hyogo | 46.5 | 42.9 | 43.7 | 44.4 |
| Nara | 45.6 | 46.6 | 47.1 | 47.7 |
| Wakayama | 37.6 | 37.4 | 37.0 | 38.4 |
| Tottori | 55.5 | 57.7 | 52.7 | 58.8 |
| Shimane | 49.4 | 41.2 | 47.8 | 50.4 |
| Okayama | 49.0 | 46.2 | 48.6 | 53.5 |
| Hiroshima | 44.4 | 43.2 | 43.2 | 47.5 |
| Yamaguchi | 33.9 | 36.4 | 38.2 | 38.4 |
| Tokushima | 44.4 | 39.7 | 43.1 | 43.8 |
| Kagawa | 39.8 | 42.8 | 44.7 | 45.0 |
| Ehime | 43.8 | 46.1 | 40.9 | 47.3 |
| Kochi | 42.0 | 38.0 | 40.2 | 40.4 |
| Fukuoka | 49.0 | 46.7 | 50.3 | 48.9 |
| Saga | 48.0 | 51.0 | 47.0 | 46.1 |
| Nagasaki | 44.0 | 44.9 | 38.8 | 39.5 |
| Kumamoto | 52.7 | 48.2 | 45.4 | 45.6 |
| Oita | 51.5 | 49.1 | 47.6 | 51.1 |
| Miyazaki | 43.6 | 41.1 | 44.5 | 43.3 |
| Kagoshima | 47.3 | 45.2 | 47.0 | 45.1 |
| Okinawa | 68.3 | 66.6 | 67.5 | 63.2 |

Missing data for 2017 is due to anonymisation.

Supplementary Table 5 Prefectural number of medical specialists in orthopaedics, general surgery, and anaesthesiology during 2015–2019 (per 100,000 inhabitants)

|  | Orthopaedic surgeon specialist | | General surgeon specialist | | Anaesthesiology specialist | |
| --- | --- | --- | --- | --- | --- | --- |
| Year | 2016 | 2018 | 2016 | 2018 | 2016 | 2018 |
| National average | 14.2 | 14.5 | 17.3 | 17.9 | 5.8 | 6.0 |
| Name of the prefecture |  |  |  |  |  |  |
| Hokkaido | 12.9 | 13.8 | 16.1 | 17.2 | 7.7 | 8.2 |
| Aomori | 12.5 | 12.6 | 15 | 15.7 | 4.6 | 4.9 |
| Iwate | 9.7 | 10.2 | 14 | 15 | 3.7 | 3.8 |
| Miyagi | 12.1 | 12.3 | 18.9 | 19.9 | 5.4 | 5.4 |
| Akita | 13.4 | 14.2 | 16.6 | 17.2 | 3.6 | 3.7 |
| Yamagata | 15.4 | 15.7 | 15.1 | 14.8 | 3.7 | 4.6 |
| Fukushima | 11.3 | 11.1 | 14.6 | 16.1 | 3.9 | 4.3 |
| Ibaraki | 9.8 | 9.8 | 13.5 | 14.2 | 4.3 | 4.4 |
| Tochigi | 10.5 | 11.1 | 17.2 | 18.2 | 5.5 | 5 |
| Gumma | 13.2 | 14.2 | 16.8 | 17.3 | 6.1 | 6.4 |
| Saitama | 8.4 | 8.6 | 11.9 | 13 | 4 | 4.3 |
| Chiba | 10.6 | 10.9 | 15.5 | 16.4 | 4.2 | 4.4 |
| Tokyo | 13.4 | 13.8 | 20.8 | 21.1 | 7.2 | 7.5 |
| Kanagawa | 11.3 | 11.7 | 13.8 | 14.4 | 5.3 | 5.4 |
| Niigata | 12.6 | 12.6 | 11.7 | 12 | 3.3 | 3.9 |
| Toyama | 15.5 | 15.3 | 16.6 | 16.6 | 6.7 | 6.7 |
| Ishikawa | 15.2 | 15.7 | 19.5 | 19.4 | 6.3 | 7.3 |
| Fukui | 15.1 | 15.5 | 18.7 | 19.1 | 5.2 | 5.6 |
| Yamanashi | 15.3 | 15.1 | 17.6 | 18.4 | 5.1 | 5.9 |
| Nagano | 12.9 | 13.2 | 16 | 16.7 | 5.8 | 5.5 |
| Gifu | 11.6 | 11.8 | 12.9 | 14 | 4 | 3.8 |
| Shizuoka | 10.6 | 11.6 | 15.3 | 15.7 | 4.1 | 4.4 |
| Aichi | 11.2 | 11.1 | 13.6 | 13.9 | 4 | 4.3 |
| Mie | 12.7 | 13.5 | 16.3 | 15.9 | 3.3 | 3.4 |
| Shiga | 12.5 | 12.1 | 15 | 14.6 | 5.6 | 5.9 |
| Kyoto | 16.8 | 17.4 | 25.5 | 25.3 | 7.4 | 7.7 |
| Osaka | 14.2 | 15.1 | 18.9 | 19.4 | 6.4 | 6.9 |
| Hyogo | 14.4 | 14.6 | 16.8 | 17.8 | 5.9 | 6 |
| Nara | 17.6 | 18 | 16.6 | 17.8 | 5.4 | 5.8 |
| Wakayama | 16.8 | 17.8 | 18.1 | 19.7 | 6.1 | 6.5 |
| Tottori | 16.5 | 17 | 20.4 | 20 | 6.8 | 6.8 |
| Shimane | 14.8 | 15.1 | 18.1 | 19.3 | 8.4 | 8.5 |
| Okayama | 15 | 15.6 | 21.1 | 21.4 | 7.2 | 7.9 |
| Hiroshima | 15.7 | 15.7 | 19.9 | 20 | 6.1 | 6.1 |
| Yamaguchi | 14.6 | 14.2 | 17.6 | 18.9 | 6.3 | 6.1 |
| Tokushima | 17.2 | 18.5 | 18.8 | 18.1 | 7.5 | 6.7 |
| Kagawa | 18.9 | 18.9 | 18.1 | 20.1 | 7.2 | 7.2 |
| Ehime | 16.1 | 16.1 | 19.2 | 19.6 | 6.1 | 6.4 |
| Kochi | 19 | 18 | 20.7 | 21.2 | 6.8 | 7.5 |
| Fukuoka | 17.4 | 17.8 | 21.4 | 22.9 | 6.9 | 7 |
| Saga | 18.8 | 18.9 | 17 | 18.7 | 7.1 | 7 |
| Nagasaki | 16.9 | 17.3 | 19.7 | 20.3 | 6.3 | 6.8 |
| Kumamoto | 18.2 | 19 | 18.2 | 19.2 | 7.4 | 7.4 |
| Oita | 15.9 | 15.4 | 21.1 | 22.6 | 7.1 | 7.3 |
| Miyazaki | 17.1 | 16.6 | 18.1 | 19.1 | 7.4 | 7.7 |
| Kagoshima | 15 | 15.1 | 17.8 | 19.2 | 7 | 7.4 |
| Okinawa | 11.5 | 11.7 | 16.1 | 16.2 | 6.1 | 6.8 |

Supplementary Table 6 Prefectural number of medical facilities (hospitals and beds) during 2015–2019 (per 100,000 inhabitants for hospitals and per 1,000 inhabitants for beds)

|  | Hospitals | Beds |
| --- | --- | --- |
| Year | 2017 | 2017 |
| National average | 8.1 | 1,412 |
| Name of the prefecture |  |  |
| Hokkaido | 10.5 | 1,777 |
| Aomori | 7.4 | 1,350 |
| Iwate | 7.4 | 1,379 |
| Miyagi | 6.0 | 1,100 |
| Akita | 6.9 | 1,512 |
| Yamagata | 6.3 | 1,324 |
| Fukushima | 6.8 | 1,357 |
| Ibaraki | 6.1 | 1,092 |
| Tochigi | 5.5 | 1,078 |
| Gumma | 6.6 | 1,236 |
| Saitama | 4.7 | 853 |
| Chiba | 4.6 | 953 |
| Tokyo | 4.7 | 935 |
| Kanagawa | 3.7 | 806 |
| Niigata | 5.7 | 1,253 |
| Toyama | 10.0 | 1,575 |
| Ishikawa | 8.2 | 1,561 |
| Fukui | 8.7 | 1,401 |
| Yamanashi | 7.3 | 1,317 |
| Nagano | 6.2 | 1,150 |
| Gifu | 5.0 | 1,019 |
| Shizuoka | 4.9 | 1,052 |
| Aichi | 4.3 | 899 |
| Mie | 5.4 | 1,121 |
| Shiga | 4.0 | 1,016 |
| Kyoto | 6.5 | 1,359 |
| Osaka | 5.9 | 1,212 |
| Hyogo | 6.4 | 1,182 |
| Nara | 5.9 | 1,258 |
| Wakayama | 8.8 | 1,426 |
| Tottori | 7.8 | 1,513 |
| Shimane | 7.4 | 1,541 |
| Okayama | 8.5 | 1,480 |
| Hiroshima | 8.6 | 1,412 |
| Yamaguchi | 10.5 | 1,931 |
| Tokushima | 14.7 | 1,942 |
| Kagawa | 9.2 | 1,537 |
| Ehime | 10.3 | 1,611 |
| Kochi | 18.1 | 2,545 |
| Fukuoka | 9.0 | 1,672 |
| Saga | 12.9 | 1,818 |
| Nagasaki | 11.1 | 1,942 |
| Kumamoto | 12.1 | 1,962 |
| Oita | 13.6 | 1,737 |
| Miyazaki | 12.9 | 1,755 |
| Kagoshima | 15.1 | 2,073 |
| Okinawa | 6.5 | 1,316 |

Supplementary Table 7 Prefectural index of the ageing rate during 2015–2019

| Year | 2015 | 2016 | 2017 | 2018 | 2019 |
| --- | --- | --- | --- | --- | --- |
| National average | 0.267 | 0.272 | 0.277 | 0.281 | 0.284 |
| Name of the prefecture |  |  |  |  |  |
| Hokkaido | 0.291 | 0.299 | 0.307 | 0.313 | 0.319 |
| Aomori | 0.301 | 0.311 | 0.319 | 0.326 | 0.333 |
| Iwate | 0.304 | 0.312 | 0.319 | 0.326 | 0.331 |
| Miyagi | 0.257 | 0.264 | 0.271 | 0.278 | 0.284 |
| Akita | 0.339 | 0.348 | 0.356 | 0.365 | 0.373 |
| Yamagata | 0.308 | 0.314 | 0.323 | 0.329 | 0.335 |
| Fukushima | 0.287 | 0.295 | 0.302 | 0.309 | 0.316 |
| Ibaraki | 0.267 | 0.276 | 0.283 | 0.289 | 0.294 |
| Tochigi | 0.259 | 0.267 | 0.274 | 0.280 | 0.286 |
| Gumma | 0.276 | 0.283 | 0.289 | 0.294 | 0.298 |
| Saitama | 0.248 | 0.255 | 0.260 | 0.264 | 0.267 |
| Chiba | 0.259 | 0.266 | 0.271 | 0.275 | 0.278 |
| Tokyo | 0.227 | 0.229 | 0.230 | 0.231 | 0.230 |
| Kanagawa | 0.239 | 0.244 | 0.248 | 0.251 | 0.253 |
| Niigata | 0.298 | 0.306 | 0.313 | 0.319 | 0.324 |
| Toyama | 0.306 | 0.312 | 0.316 | 0.320 | 0.323 |
| Ishikawa | 0.279 | 0.284 | 0.289 | 0.292 | 0.296 |
| Fukui | 0.287 | 0.292 | 0.299 | 0.304 | 0.306 |
| Yamanashi | 0.284 | 0.290 | 0.298 | 0.304 | 0.308 |
| Nagano | 0.301 | 0.307 | 0.312 | 0.315 | 0.319 |
| Gifu | 0.281 | 0.287 | 0.294 | 0.298 | 0.301 |
| Shizuoka | 0.278 | 0.285 | 0.291 | 0.295 | 0.299 |
| Aichi | 0.238 | 0.243 | 0.246 | 0.249 | 0.251 |
| Mie | 0.279 | 0.285 | 0.289 | 0.295 | 0.298 |
| Shiga | 0.243 | 0.248 | 0.253 | 0.258 | 0.260 |
| Kyoto | 0.276 | 0.281 | 0.285 | 0.289 | 0.292 |
| Osaka | 0.262 | 0.268 | 0.272 | 0.275 | 0.276 |
| Hyogo | 0.271 | 0.278 | 0.283 | 0.288 | 0.291 |
| Nara | 0.287 | 0.296 | 0.303 | 0.309 | 0.314 |
| Wakayama | 0.308 | 0.316 | 0.323 | 0.327 | 0.331 |
| Tottori | 0.298 | 0.304 | 0.312 | 0.316 | 0.320 |
| Shimane | 0.326 | 0.332 | 0.336 | 0.340 | 0.343 |
| Okayama | 0.287 | 0.293 | 0.297 | 0.301 | 0.303 |
| Hiroshima | 0.275 | 0.282 | 0.286 | 0.290 | 0.293 |
| Yamaguchi | 0.321 | 0.328 | 0.334 | 0.339 | 0.343 |
| Tokushima | 0.311 | 0.317 | 0.324 | 0.330 | 0.337 |
| Kagawa | 0.299 | 0.308 | 0.312 | 0.316 | 0.319 |
| Ehime | 0.307 | 0.313 | 0.321 | 0.325 | 0.329 |
| Kochi | 0.328 | 0.336 | 0.343 | 0.348 | 0.352 |
| Fukuoka | 0.259 | 0.266 | 0.271 | 0.276 | 0.279 |
| Saga | 0.277 | 0.284 | 0.292 | 0.298 | 0.302 |
| Nagasaki | 0.297 | 0.305 | 0.312 | 0.320 | 0.326 |
| Kumamoto | 0.288 | 0.295 | 0.301 | 0.306 | 0.310 |
| Oita | 0.304 | 0.312 | 0.319 | 0.324 | 0.329 |
| Miyazaki | 0.296 | 0.304 | 0.310 | 0.316 | 0.323 |
| Kagoshima | 0.295 | 0.301 | 0.308 | 0.314 | 0.320 |
| Okinawa | 0.197 | 0.204 | 0.211 | 0.216 | 0.222 |
